# The development prospection of HDAC inhibitors as a potential therapeutic direction in Alzheimer’s disease

**DOI:** 10.1186/s40035-017-0089-1

**Published:** 2017-07-10

**Authors:** Shuang-shuang Yang, Rui Zhang, Gang Wang, Yong-fang Zhang

**Affiliations:** 1 Department of Pharmacology, Institute of Medical Sciences, School of Medicine, Shanghai Jiao Tong University, 280 South Chongqing Road, Shanghai, 200025 China; 2 Department of Neurology Ruijin Hospital, School of Medicine, Shanghai Jiao Tong University, Ruijin 2nd Road 197, Shanghai, 200025 China

**Keywords:** Histone deacetylase inhibitors, Alzheimer’s disease, Side effects, Specificity, Efficacy

## Abstract

Alzheimer’s disease (AD) is a chronic neurodegenerative disease, which is associated with learning and memory impairment in the elderly. Recent studies have found that treating AD in the way of chromatin remodeling via histone acetylation is a promising therapeutic regimen. In a number of recent studies, inhibitors of histone deacetylase (HDACs) have been found to be a novel promising therapeutic agents for neurological disorders, particularly for AD and other neurodegenerative diseases. Although HDAC inhibitors have the ability to ameliorate cognitive impairment, successful treatments in the classic AD animal model are rarely translated into clinical trials. As for the reduction of unwanted side effects, the development of HDAC inhibitors with increased isoform selectivity or seeking other directions is a key issue that needs to be addressed. The review focused on literatures on epigenetic mechanisms in recent years, especially on histone acetylation in terms of the enhancement of specificity, efficacy and avoiding side effects for treating AD.

## Background

Alzheimer’s disease (AD) is a common form of chronic neurodegenerative dementia which is characterized by cognitive impairment and memory deficits [1]. At present, although AD is the most common type of brain disorder caused by multiple factors, its etiology and pathogenesis haven’t been fully understood. The major hallmarks of AD pathogenesis, amyloid-β(Aβ) plaques and tau neurofibrillary tangles (NFTs), may cause synaptic loss [2]. The abnormal aggregation and deposition of Aβ are neurotoxic and further cause the pathological changes of the cerebral cortex and apoptosis of nerve cells. NFTs are composed of double helix fibers produced by abnormal phosphorylation of tau protein, which leads to cell death by disrupting transportation. As a complex and interactive relationship between genes and environment, it is believed that epigenetic mechanisms including histone acetylation, DNA methylation, and microRNA modification, are also vital factors to the pathology of AD [3].

Epigenetic modification has opened up a new way for the study of AD. DNA methylation and histone modification have become one of the most significant research hot spots. In previous studies, a great deal of evidence has indicated that histone acetylation plays a vital role in rescuing learning and memory impairment [4]. Histones are building blocks of the nucleosome which is the fundamental unit of chromatin. It is the joint action and dynamic equilibrium of histone-acetyltransferases (HATs) and histone-deacetylases (HDACs) that regulate histone acetylation properly [5]. In general, histone acetylation makes chromatin more loosely packed to activate transcription while histone-deacetylation represses gene transcription.

The state of chromatin structure depends largely on the chemical modification of histone protein complexes. In addition to methylation, phosphorylation, sulfonylation, and ADP ribosylation, histone acetylation serves as the essential role in transcriptional regulation [6]. In normal neurons, the protein levels of HAT and HDAC as well as their corresponding activities keep in a highly-harmonized balance state, which is conductive to regulate normal gene expression and neuro-physiological outputs. However, the acetylation homeostasis is disturbed in neurodegenerative state [7]. Histone deacetylase inhibitors (HDACIs) have, therefore, gained increasing attention and interest as a promising treatment alternative for the field of neurodegenerative diseases [8, 9].

In AD animal models, HDAC inhibitors exhibit neuroprotective and neurodegenerative properties, and it is a promising strategy for brain diseases [10]. Most of the HDAC inhibitors that treat AD models are poorly selective and often cause some undesirable side effects. Therefore, comprehending the position of individual HDAC in AD pathogenesis is critical to the development of more selective HDAC inhibitors. Researchers are looking for various strategies to avoid side effects as much as possible.

Based on the findings in recent years, in this review, we summarized methods to reduce side effects and improve drug efficacy, and some issues remained to be addressed in the future.

## Histone deacetylase inhibitors

Theoretically speaking, HDAC inhibitors were originally applied to the cancer therapy. In 2008, Hahnen and colleagues found that the histone deacetylase inhibitors were possible to be an effective strategy for neurodegenerative disorders and identified two major neuroprotective mechanisms including the transcriptional activation of disease-modifying genes and the rectification of destabilization in histone acetylation homeostasis [11].

Recently, many studies have proved that histone acetylation is reduced in various neurodegenerative disorders, such as AD [12]. For example, treatment of SH-SY5Y cells with trichostatin A, a HDAC inhibitor, resulted in a marked up-regulation of Aβ-degrading enzyme neprilysin (NEP) expression [13]. Administration of histone deacetylase inhibitor MS-275 in APP/PS1 models showed ameliorated microglial activation, decreased Aβ deposition, and attenuated inflammatory activation in vitro as well [14]. Therefore, the study of increasing histone acetylation to ameliorate the impairment of memory in AD patients may be a promising strategy. Generally, histone acetylation usually activates gene transcription, whereas histone deacetylation is closely associated with gene transcriptional repression [15]. Both of them alter chromatin structure and transcription factors for the regulation of gene expression.

## Strategies to reduce side effects

### Enhancing specificity of targeted HDAC isoforms to reduce side effects

Studies have shown that HDAC inhibitors exhibit neuroprotective properties in AD mouse models. Nevertheless, the action of specific HDACs in neurodegenerative disease remains poorly understood in detail and merely pan-HDAC inhibitors were tested in preclinical studies so far. Identifying the role of individual HDAC in AD is critical to the development of specific HDAC inhibitors in curing AD.

In mammals, HDAC enzymes are sorted out into four classes on the basis of their homology to yeast. Class I of HDACs is mainly located in the nucleus and, contains HDAC 1, 2, 3, and 8. Class II of HDACs is further separated into two subtypes: Class IIa includes HDAC4, 5, 7, 9; class IIb includes HDAC 6, 10. In general, class III of HDACs is called as “sirtuins”. Class III of HDACs sharing homologous sequence with Sir2 protein contains SirT1-7. Class IV HDACs only has HDAC11 that possesses a catalytic domain at the N-terminus [1].

It is believed that unfavorable side effects are associated with pan-selective isoforms. Enhancing the specificity of isoforms of HDAC inhibitors may be more effective and beneficial to restore cognitive deficits in the AD models.

#### Targeting at class I of HDACs to enhance specificity

It is the isoform selectivity that determines the specific properties of class I HDAC inhibitors to ameliorate memory impairment. The location and levels of expression differ according to the individual form of different HDACs. Compared with HDAC2 and −3, HDAC1 were detected at relatively lower mRNA levels [16]. A novel selective HDAC2 inhibitor called as W2 was found to decrease Aβ levels and further ameliorate cognitive impairment via promoting the formation and growth of dendric spine density [8, 17].

It’s known that amyloid-oligomer attenuates long-term potentiation (LTP), RGFP966 is a specific HDAC3 inhibitor that is a crucial epigenetic negative regulator of cognition. Kumar Krishna et al. provided the first evidence that inhibiting HDAC3 enzyme in single neuron or neuronal populations had the ability of preventing synaptic plasticity induced by amyloid-oligomer [18].

Furthermore, in terms of stimulating synaptogenesis, RGFP963 and RGFP968, acted more efficiently as the HDAC inhibitors comparing with RGFP966, a selective HDAC inhibitor with the ability of ameliorating cognition deficits. Additionally, RGFP963 was invalid in inhibiting HDAC3 while could increase hippocampal spine density. Subsequent research indicated that RGFP963 rather than RGFP966 induced a transcriptional mechanism that enhanced synaptic efficacy and finally rescued learning and memory abilities in AD mouse models [16]. Consistent with the results, researches indicated that inhibiting class I HDAC isoforms simultaneously could be an promising therapy, which might bring a transcriptional synergy and further increase efficacy of producing synapses and treating cognition impairment [16].

#### Targeting at class IIa of HDACs to enhance specificity

However, not every isoform can be the target to inhibit and ameliorate pathological state. Take HDAC5 for example, R. C. Agis-Balboa et al. tested the role of HDAC5 in AD pathogenesis. They observed the phenomenon that reduced HDAC5 could cause memory impairment and rarely affect the deposition of amyloid plaque [19]. This study elucidated a special function of HDAC5 and revealed HDAC5 isoform should be excluded when seeking for selective HDAC inhibitors in further approaches aiming at treating AD.

#### Targeting at class IIb of HDACs to enhance specificity

Reduction of HDAC6 is associated with the clearance of both tau and Aβ [20, 21]. HDAC6 is a vital regulator for mitochondrial transport and pharmacological inhibition of HDAC6 promotes the mitochondrial dynamics in Aβ-treated neurons, which can be identified as a new the therapeutic approach [22]. Most importantly, inhibition of HDAC6 is not harmful for cell survival [23]. Inhibiting HDAC6 via deacetylating α-tubulin significantly restored the length of the mitochondria shortened by Aβ to a normal level and rescued hippocampal neuron impairment induced by Aβ. Similarly, one study found that the loss of endogenous HDAC6 levels restored cognitive dysfunction and α-tubulin acetylation in AD mouse models [24]. The finding revealed the therapeutic effect was associated with reduction of HDAC6 rendered neurons against Aβ-mediated disorder of mitochondrial trafficking [24]. HDAC6 might be developed as a potential suitable target for treating AD.

Research found that two selective HDAC6 inhibitors, tubastatin A and ACY-1215, could improve microtubule stability and ameliorate cognitive impairment in AD mouse by promoting tubulin acetylation, reducing production of Aβ and hyper-phosphorylated tau and facilitating autophagic clearance of Aβ and hyper-phosphorylated tau [25]. Compared with SAHA, tubastatin A and ACY-1215 are less toxic indeed. These preclinical results offer prospective approaches for the application of tubastatin A/ACY-1215 to cure AD.

In addition, HDAC6 can drive the deacetylation of KXGS motifs, and further promotes hyper-phosphorylation and polymerization of tau. On the basis of this pathogenic mechanism, inhibiting HDAC6 selectively could improve acetylation and prevent tau accumulation. According to this finding, HDAC6 inhibitors have the ability of enhancing acetylation on KXGS motifs by shifting the ratio between acetylation and phosphorylation to alleviate tau burden. In brief, inhibitors that focus on HDAC6 alone might be a potential perspective for AD with less side effect [26].

### Drug combination to reduce side effects

Pan-HDAC inhibitors usually contain unwanted off-target effects limiting the clinical application unavoidably. Drug combination is a feasible strategy that could utilize the compensatory mechanisms and overcome the disadvantages [27]. Indeed, simultaneously inhibiting different targets is possible to bring more efficient effects compared with only use of specific inhibitors [28]. Acting on several effective enzyme activities simultaneously is also a novel pattern of inhibitory action that can reduce side effect. Combining vorinostat (a pan-HDACI) with tadalafil (a phosphodiesterase-5 inhibitor), cognitive deficits, LTP as well as the amyloid and tau pathology were alleviated in AD mice. Remarkably, take curative effect and duration of drug action into account, combining vorinostat with tadalafil was much better than each drug alone [29].

Moreover, Suberoylanilide Hydroxamic Acid (SAHA) is a HDAC inhibitor that can improve cognition [30]. But SAHA is a non-selective inhibitor owing to extensive target at HDAC 1, 2, 3, 6, 8 [31]. Experiments showed that combination of low doses SAHA and curcumin could improve therapeutic selectivity and provide comprehensive protection against Aβ-induced neuronal deficits [32]. These synergisms may contribute to the realization of more optimal safety profiles for HDACIs in chronic treatments.

### Balanced dual inhibitors to reduce side effects

To develop a selective and potent drug with minimal unwanted off-target effects, a new attempt is to design selective chemical probes possessing unequivocally validate targets. This method requires chemical probes with optimizing pharmacokinetics so that they could cross the blood brain barrier (BBB) and simultaneously meet the critical safety criteria. These probes will be essential pharmacological tools for in vivo target validation in the search for AD pharmacotherapies [33]. In 2017, Mar Cuadrado-Tejedor and colleagues discovered a new first-in-class small-molecule (CM-414) which is a dual inhibitor of PDE5 and HDACs [34]. CM-414 acts as a chemical probe to achieve both moderate class I HDAC inhibition and PDE5 inhibition that makes histone acetylation more efficient. CM-414 significantly ameliorated the impaired (LTP) and reduced the levels of brain Aβ and tau phosphorylation in Tg2576 mice. As a balanced dual inhibitor, CM-414 may offer an innovative originality to find methods that provided safe and effective treatment for AD patients.

## Strategies to improve drug efficacy

### Co-location to improve drug efficacy

Not only AD treatment, but the therapies of other aging disease are associated with HDACIs. To receive multiple effects, one study observed that HDAC2 was co-located with insulin signaling components in postsynaptic glutamatergic neurons (PSGNs) of the mouse hippocampus. This may be conductive to understand the roles of both HDAC2 and insulin on neurological deficits of diabetes mellitus, in relation to AD. Of course, it is also beneficial to treat insulin-related diseases when referring to the cognition [35]. This method is available for those patients who suffered from multi-aging diseases.

### Increasing permeability to improve drug efficacy

Two novel HDAC inhibitors, mercaptoacetamide-based class II HDACI (represented by W2) and hydroxyamide-based class I and II HDACI (represented by I2), were developed recently and both could reduce Aβ levels obviously and restored cognitive impairment. Most significantly, both W2 and I2 could go through blood brain barrier (BBB) more easily and have a longer half-life [17]. Side effects could be reduced by prolonging half-life and increasing permeability to BBB, which are beneficial to extend plasma half-life of drugs, elevate plasma drug concentration, increase therapeutic effect and diminish baneful adverse reaction.

### Stage treatment to improve drug efficacy

Additionally, HDAC inhibitors need to be considered in age-dependent and/or stage-dependent manner when representing as a therapeutic target for AD. The study by Noh H et al. classified the pathogenic process into three different disease stages and sought therapeutic regimen from various angles in detail. They found administration of valproic acid (VPA) to inhibit HDAC reduced cytokine expression levels, raised sAPP and nerve growth factor (NGF) and finally enhanced cognitive function in Tg6799 AD mice [36]. AD in different progress stages may produce specific markers and stage-intervention targeted therapy would be a optimal choice.

### Drug administration routes to improve drug efficacy

In recent years, apart from the isoforms-specific of HDAC inhibitors have been significantly developed, what is the best dosing regimen for HDACI to produce neuroprotection, that is, long term treatment vs pulse treatment vs acute treatment is still unclear. Similar to broad-spectrum HDACIs, dose-dependent side effects in patients is the reason of the restriction for chronic disease. Pulse treatment with trichostatin-A (TSA) was shown histone hyperacetylation and neuroprotection in previous study. Post-occlusion administration of sodium butyrate (SB) ameliorates cognitive impairment in a rat model induced by chronic cerebral hypo-perfusion (CCH) [37]. To some extent, treatment strategies like these may get long term HDACI drug treatment away from toxicity and side effects. In addition, HDAC inhibitors combined with other neuroprotective agents may be a good treatment regimen. HDAC inhibitors perhaps could be considered as an ideal target in combination with other neuroprotectants.

## Conclusions and perspective

Given the increasing AD patients due to the extension of life span, and the lack of effective treatments, it is urgent to develop novel potential drugs. Most HDAC inhibitors are pan-targeted and easily cause more off-targeted side effects. Successful treatments in the classic AD animal model are also rarely translated into clinical trials, therefore further work is required to validate the findings detailed above before clinical use. Accordingly, therapeutic specificity for neurodegenerative disease can be improved by increasing HDACI isoform selectivity that has the benefit to simultaneously decrease off-target effects [38]. Besides, most researchers have to explore the link between various HDAC isoforms and cognition, particularly the cognitive impairment in mouse models. Enhancing isoform selectivity of HDAC inhibitors is the congruence of goals shared by those who intend to reduce unwanted side effects.

Many research institutes are actively working on selective, specific and orally effective HDAC inhibitors. The inhibitors that are able to enhance HDAC isoform specificity and pharmaceutical potency are thought to possess new chemical scaffolds and have received extensive concern. Researchers have been paying closer attention to the emerging medicinal chemistry principles of HDAC inhibitors and shifting trial-and-error approaches to sophisticated strategies to promote the discovery of ideal-effective HDAC inhibitors [39]. The development of HDACIs with increased isoform selectivity or seeking other directions in order to reduce unwanted side effects is a key issue to be addressed. Based on the various issues that hinder the use of HDAC inhibitors in clinical setting, this review is to sum up the recent research based on the various issues of HDAC inhibitors for reducing defects and improvingspecificity/efficacy. However, current articles on selective HDAC inhibitors are not enough to address the clinical application and much work remains to be carried out in the future.

## Abbreviations

AD: Alzheimer’s disease
Aβ: amyloid-β
BBB: Blood brain barrier
CCH: Chronic cerebral hypo-perfusion
HATs: Histone acetyltransferases
HDACIs: Histone deacetylases inhibitors
HDACs: Histone deacetylases
LTP: Long-term potentiation
NEP: Neprilysin
NFTs: Neurofibrillary tangles
NGF: Nerve growth factor
SB: Sodium butyrate
